# Micro- and Macrovascular Effects of Inflammation in Peripheral Artery Disease—Pathophysiology and Translational Therapeutic Approaches

**DOI:** 10.3390/biomedicines11082284

**Published:** 2023-08-17

**Authors:** Michael Poledniczek, Christoph Neumayer, Christoph W. Kopp, Oliver Schlager, Thomas Gremmel, Alicja Jozkowicz, Michael E. Gschwandtner, Renate Koppensteiner, Patricia P. Wadowski

**Affiliations:** 1 Division of Angiology, Department of Internal Medicine II, Medical University of Vienna, 1090 Vienna, Austria; michael.poledniczek@meduniwien.ac.at (M.P.); christoph.kopp@meduniwien.ac.at (C.W.K.); oliver.schlager@meduniwien.ac.at (O.S.); michael.gschwandtner@meduniwien.ac.at (M.E.G.); renate.koppensteiner@meduniwien.ac.at (R.K.); 2 Division of Cardiology, Department of Internal Medicine II, Medical University of Vienna, 1090 Vienna, Austria; 3 Division of Vascular Surgery, Department of General Surgery, Medical University of Vienna, 1090 Vienna, Austria; christoph.neumayer@meduniwien.ac.at; 4 Department of Internal Medicine I, Cardiology and Intensive Care Medicine, Landesklinikum Mistelbach-Gänserndorf, 2130 Mistelbach, Austria; thomas.gremmel@mistelbach.lknoe.at; 5 Institute of Cardiovascular Pharmacotherapy and Interventional Cardiology, Karl Landsteiner Society, 3100 St. Pölten, Austria; 6 Department of Medical Biotechnology, Faculty of Biophysics, Biochemistry and Biotechnology, Jagiellonian University, 31-007 Krakow, Poland; alicja.jozkowicz@uj.edu.pl

**Keywords:** atherosclerosis, inflammation, peripheral artery disease, glycocalyx, endothelial dysfunction

## Abstract

Inflammation has a critical role in the development and progression of atherosclerosis. On the molecular level, inflammatory pathways negatively impact endothelial barrier properties and thus, tissue homeostasis. Conformational changes and destruction of the glycocalyx further promote pro-inflammatory pathways also contributing to pro-coagulability and a prothrombotic state. In addition, changes in the extracellular matrix composition lead to (peri-)vascular remodelling and alterations of the vessel wall, e.g., aneurysm formation. Moreover, progressive fibrosis leads to reduced tissue perfusion due to loss of functional capillaries. The present review aims at discussing the molecular and clinical effects of inflammatory processes on the micro- and macrovasculature with a focus on peripheral artery disease.

## 1. Introduction

Cardiovascular disease (CVD) is the leading cause of mortality worldwide and accounts for millions of deaths globally [1]. CVD is associated with a significant impairment of quality of life and the prevalence of its main manifestations, such as coronary artery disease (CAD), cerebrovascular disease and peripheral artery disease (PAD), has been increasing steadily over the last two decades [1,2].

Atherosclerosis is considered the major driver of CVD. Formerly, atherosclerosis was thought of as a process primarily related to dyslipidaemia and the deposition of triglycerides and cholesterol [3]. However, besides lipid accumulation, more recent insights into the pathogenesis of atherosclerosis increasingly emphasise the role of inflammation and endothelial dysfunction as major drivers of atherogenesis [3,4,5,6,7,8]. Moreover, the mentioned pathomechanisms depend on each other and amplify each other’s responses. Indeed, a central element initiating prothrombotic processes and herein, atherogenesis, remains glycocalyx destruction due to inflammatory processes [9]. In PAD, inflammation is also triggered by ischaemia-reperfusion (I/R) injury promoting increased production of reactive oxygen species (ROS) [10], which contribute to endothelial dysfunction and microvascular pathology [11].

Chronic autoimmune diseases, which are associated with significantly elevated levels of systemic inflammation, e.g., rheumatoid arthritis, systemic lupus erythematosus, anti-phospholipid syndrome and antineutrophil cytoplasmic antibody (ANCA)-associated vasculitis (AAV), are associated with markedly increased prevalence of CVD [12,13,14]. Conversely, there is accumulating evidence that some agents with anti-inflammatory characteristics reduce cardiovascular risk significantly [15,16]. Canakinumab, a monoclonal antibody targeting interleukin (IL)-1β [15], and colchicine, which attenuates leukocyte responsiveness by inhibition of tubulin polymerisation [16,17], have been shown to improve outcomes in CAD in randomized controlled trials [15,16]. While not yet implemented in regular clinical practice, there is increasing awareness for anti-inflammatory therapy in secondary prevention of CVD in the current guidelines [18].

The single most effective prevention of CVD, smoking cessation, lowers levels of systemic inflammation as assessed utilising biomarkers of inflammation and oxidative damage [19,20]. Statins, which are the most widely established agents for lipid control, also have been shown to exert significant immuno-modulatory influence by inhibiting the nuclear factor kappa B (NF-ĸB) pathway and decreasing the expression of toll-like receptors (TLR) [21]. Smoking cessation and statins are both recommended in all patients with CVD [22].

Formation of aneurysms is also considered to be linked to atherosclerosis. Recently, the role of leukocytes and, especially, neutrophils in the development of aneurysms has been revisited [23,24]. Activation of matrix metalloproteinases (MMP), degradation of the extracellular matrix (ECM), smooth muscle apoptosis and oxidative stress all contribute to aneurysm formation and are mediated by cytokines secreted by leukocytes [23,25]. Interestingly, atherosclerosis and aneurysm formation do not always occur at the same locations. While the abdominal aorta, an area of predilection for aneurysm formation, is also prone to atherosclerosis, the external iliac artery, a common location for significant atherosclerosis, is very seldomly involved in the formation of aneurysms. Which cellular and non-cellular processes discern these two locations is currently unclear, however, the different embryologic origin of these vessels may be responsible for varying susceptibility to atherosclerosis and aneurysm formation, respectively [24].

This review aims to describe inflammatory pathomechanisms implicated in atherosclerotic processes of the macro- and microvasculature, their determinants and implications for interactions with the endothelium, leukocytes and non-cellular components involved in vascular homeostasis. In addition, therapeutic applications of anti-inflammatory concepts for the management of PAD are discussed.

## 2. Pathophysiology

### 2.1. Inflammation and Endothelial Dysfunction

Endothelial and vessel homeostasis is to a wide extent ensured by an intact glycocalyx coverage [26]. The endothelial glycocalyx is located at the luminal side of the cells and consists of membrane-bound proteoglycans and, together with adsorbed proteins, forms the endothelial surface layer [27]. Its components exert significant influence on the interactions between the blood and the endothelium, including rolling and diapedesis of leukocytes [28], platelet adhesion and activation [29], interaction with pro-coagulatory proteins [27], endothelial permeability [30] and the regulation of vascular tone [31].

Dysfunction and degradation of the endothelial glycocalyx allows low-density lipoproteins (LDLs) to accumulate in the endothelial wall [32]. Following aggregation, LDL is oxidised (oxLDL) and subsequently phagocytosed by macrophages, which transform into foam cells and thereby initiate the progressive process of atherogenesis [32]. In turn, the integrity of the endothelial glycocalyx is disturbed by vascular inflammation, therefore creating a vicious cycle of endothelial dysfunction, inflammation and progression of atherosclerosis [33].

The components of the glycocalyx also play a major role in the modulation of thromboinflammatory pathways [9,34]. Importantly, the glycocalyx barrier does not only cover endothelial cells, but functions as a protective barrier exhibiting steric and charge hindrance on blood components such as macrophages, erythrocytes, microspheres, tumour cells and microbes [35,36]. Similarly, neutrophils have been demonstrated to express syndecan-1 and syndecan-4, hyaluronan, serglycin and cluster of differentiation (CD) 44 in their surface layer [37]. These molecules are essential components of both the endothelial and the neutrophil surface layers and are thought to regulate neutrophil rolling and recruitment [37]. Modifications to the neutrophil surface layer, including shedding of the glycocalyx and formation of microvilli, are thought to regulate leukocyte behaviour by exposing receptor proteins and promoting leukocyte activation [36,38]. However, the exact interactions of the endothelial and the neutrophil surface layers remain to be completely elucidated [37].

Macrophage activation after phagocytosis may lead to macrophage extracellular trap (MET) formation, but the process might be dependent on the recognized pathogen [39,40]. On the other hand, inflammation triggers leukocyte activation, promoting neutrophil– and monocyte–platelet aggregate formation [41,42]. The process is perpetuated by ETosis and enhanced oxidative stress [43,44,45].

Moreover, activated platelets lead to a thrombin burst; thrombin is the strongest platelet agonist, mediating platelet activation via protease-activated receptors (PARs) 1 and 4 at subnanomolar concentrations [46]. Platelet aggregation through these pathways has been shown to be preserved despite adequate dual P2Y_12_ inhibition in patients with acute coronary syndromes [47]. Moreover, thrombin also activates platelets via glycoprotein Ib [48]. Further, inflammation mediates platelet activation through other alternative signalling pathways, including damage-associated signalling through TLRs [34]. Human platelets express all 10 TLR receptors [49], and related inflammatory signalling leads, amongst others, to P-selectin expression, ROS formation and enhanced platelet–neutrophil contacts [50]. Moreover, TLR-induced endothelial activation results in endothelial dysfunction [51]. The complex interplay of TLR receptor signalling pathways leads through signalling cascades via toll-interleukin-1 receptor resistance (TIR) domain-containing adaptor proteins to gene expression altering via different transcription factors, such as nuclear factor-κB (NF-κB), activator protein 1 (AP-1), nuclear factor erythroid-2-related factor 2 (NRF2), activating transcription factor 2 (ATF2) and interferon regulatory factors (IRFs) [34]. In humans, there are five TIR adaptors, namely the myeloid differentiation primary response protein 88 (MyD88), TIR domain-containing adaptor protein (TIRAP), TRIF, TRIF-related adaptor molecule (TRAM) and TIR domain sterile alpha and HEAT/Armadillo motif (SARM) [34,52,53,54,55].

All human TLRs signal via MyD88 to mediate inflammatory cytokine production [56,57]. However, NF-κB and the IRFs can be activated via MyD88-dependent, as well as MyD88-independent, pathways [53,58,59]. TLR-induced NF-κB activation modulates the NLRP3 inflammasome, which is a major mediator of IL-1 family cytokine production [60,61]. NLRP3 activation is directly involved in endothelial dysfunction, and enhanced expression was found in the serum of PAD patients [62,63].

TLR-4-mediated signalling in platelets, neutrophils and macrophages also contributes to neutrophil extracellular trap (NET) and MET formation, respectively [64,65,66,67].

Some risk factors commonly associated with atherosclerosis and thromboembolic events are also thought to impair the integrity of the glycocalyx [33]. Chronic diseases, such as diabetes mellitus (DM) and chronic kidney disease, are often linked to inflammatory processes and promote glycocalyx disturbance [33,68,69,70,71,72,73,74].

Several pathophysiologic properties link atherosclerosis and DM [75]. First, DM-associated dyslipidaemia leads to increased triglyceride-rich lipoproteins (TLP) in serum [75]. Under physiologic circumstances, insulin regulates hepatic lipoprotein and triglyceride production, however, in DM, these regulatory properties are diminished due to hepatic insulin resistance [75]. It has been demonstrated that not only the prevalence of lipoproteins, but also their modifications, can be considered essential for atherogenesis [76]. In a murine model of DM, an injection of LDL from diabetic patients resulted in a fourfold increase in arterial wall LDL retention compared to injected LDL from clinically healthy, non-diabetic control subjects [76].

Advanced glycation end-products (AGEs) are formed in patients with prolonged hyperglycaemia by non-enzymatic post-translational modification of proteins, lipids and nucleic acids [77]. AGEs promote inflammation by facilitating the activation of the endothelium, increasing cytokine release from macrophages, and ultimately, enhancing ROS production [10]. The latter are also key in I/R injury in PAD and contribute to inflammatory processes and endothelial dysfunction [10]. Ischaemia leads to succinate accumulation due to impaired mitochondrial citric acid cycle (TCA) [78,79]. Succinate can be transported to the cytosol, where, due to its excess, it leads to prolyl hydroxylase activity impairment and, in turn, to the stabilization and activation of the transcription factor hypoxia-inducible factor 1 (HIF-1) α. This pathway results in the expression of IL-1ß [80]. In addition, succinate accumulation is a hallmark of macrophage polarisation, occurring in the pro-inflammatory M1 macrophages [81].

Reperfusion leads to rapid reoxidation of succinate by succinate dehydrogenase, driving extensive ROS generation [82]. During I/R injury, NO bioavailability is decreased, and ROS activate the nucleotide oligomerization domain, leucine-rich repeat, and pyrin domain-containing protein 3 (NLRP3) inflammasomes, promote mitochondrial fission and endothelial microvesicle release, and change connexin/pannexin signalling [11]. As a result of the oxidative stress, I/R impairs capillary perfusion [11]. Furthermore, reduced NO levels promote M1 polarisation [83].

CVD including PAD is further linked to a reduced endothelial progenitor cell (EPC) number [84]. The inflammatory processes induced by uncontrolled oxidative stress also modify EPC function and thus impair endothelial regenerative potential [85]. In response to ischaemia, EPC release has been demonstrated to be markedly decreased in patients with PAD compared to healthy control subjects [86]. After mobilization, EPCs were shown to home to ischaemic tissue, facilitated by vascular growth factor (VEGF) and stromal cell-derived factor 1 (SDF-1) [87]. The latter binds to C-X-C chemokine receptor type 4 (CXCR-4) on EPCs [88].

EPCs have been shown to express gene transcripts coding for TLR 1–6, including the TLR-4 co-receptor CD14, TLR 8–10 and the TLR adaptor molecule myeloid differentiation factor 88 (MyD88) [89]. Hence, during inflammation, EPCs might also be modulated by TLR signalling pathways, such as TLR-4 mediated caspase 3 signalling promoting EPC apoptosis [85,90]. In addition, ROS formation triggers extracellular trap formation by different cells of the immune system such as neutrophils, eosinophiles, macrophages and mast cells, hereby influencing coagulability and vascular perfusion [34,91].

Coronavirus disease 2019 (COVID-19), which increases the risk of thromboembolic events during and after the infection [92], is also thought to impair the regular functioning of the glycocalyx [9,93,94]. The degradation of the glycocalyx is mediated by a complex interaction of cellular and non-cellular factors but is mainly driven by infection of endothelial cells by severe acute respiratory distress syndrome coronavirus type 2 (SARS-CoV-2) [95]. Subsequent endothelial inflammation and damage leads to disintegration of the glycocalyx, collagen exposure and, thereupon, activation of leukocytes and platelets [96]. These processes are thought to lead to an environment of thromboinflammation, which may ultimately trigger atherogenic processes and promote organ dysfunction [9,94].

### 2.2. Microparticles

Microparticles (MP) are cell-membrane-derived vesicles which are shed by, among others, endothelial cells, leukocytes, monocytes and platelets [97] at an increased rate upon cell activation due to oxidative injury, shear stress and apoptosis [98]. MPs can carry a plethora of cell-specific proteins and molecules such as receptors, lipids and both mitochondrial desoxyribonucleic acid (DNA) and messenger ribonucleic acid (mRNA) [97]. MPs are thought to contribute to cell–cell communication as their surface is representative of the originator cell [97,99,100]. Novel diagnostic and therapeutic applications are currently under investigation and first results seem promising [101]. MP composition has been demonstrated to be altered in inflammatory conditions, where endothelial cells stimulated with tumour necrosis factor (TNF)-α secrete MPs rich in pro-inflammatory cytokines and chemokines [102]. Intercellular signalling via MPs is therefore considered to exert a significant regulatory role in vascular homeostasis [103,104].

Under physiologic conditions, endothelial nitric oxide (NO) synthetase maintains vascular homeostasis by regulation of vascular tone and inhibition of platelet function through NO [105]. In addition, NO promotes anti-inflammatory M2 macrophage polarisation and limits the pro-inflammatory M1 phenotype [83]. In conditions associated with CVD, e.g., hypertension, tobacco abuse and dyslipidaemia, the endothelial production of NO is drastically reduced, leading to increased platelet activation and leukocyte diapedesis [105,106,107].

As described above, endothelial dysfunction is generally considered the earliest stage of atherogenesis [108]. While at physiological levels, ROS serve as signalling molecules with effects on cell differentiation, growth and apoptosis, in higher levels, their ability to oxidise various molecules results in cellular dysfunction and inflammation [109,110]. Under normal conditions, ROS are generated by mitochondria in the course of the electron transport chain, by xanthin oxidase and uncoupled endothelial NO synthetase (eNOS), and by nicotinamide adenine dinucleotide phosphate (NADPH) oxidase [110]. The latter is especially important as a source of ROS in host-defence responses and inflammation [109].

ROS trigger a range of cellular responses, which include the activation of the NLRP3 inflammasome and consecutive IL-1β activation, the inhibition of eNOS via peroxisome proliferator-activated receptor (PPAR)-γ and adenosine-monophosphate-kinase (AMPK), and increased expression of adhesion molecules and several pro-inflammatory cytokines [110,111,112]. ROS have also been demonstrated to activate the TLR-4-mediated NF-ĸB signalling pathway [113] and, therefore, stimulate further ROS formation [110,114]. In addition, MPs also bind to TLR-4, and can induce NLRP3 inflammasome activation and IL-1ß expression through phosphatidylinositol 3-kinase (PI3K)/protein kinase B (Akt) signalling [115]. By binding to TLR-4 on platelets, MPs also contribute to platelet activation [116].

Furthermore, MPs can aggravate ROS production by expressing NADPH oxidase [117,118], potentially creating a vicious cycle of self-sustained pro-atherogenic stimuli. Importantly, MPs can not only induce the release of pro-inflammatory cytokines and ROS, but can in fact act as a vehicle of transfer between donor and recipient cells, conferring both pro- as well as anti-inflammatory effects [119].

In particular, MPs derived from endothelial cells (EMP) and platelets (PMP) disrupt endothelial function and impair endothelium-induced vasodilation [120,121]. Formation of EMPs has been shown to correlate with carotid artery atherosclerotic plaque size in patients recovering from stroke [122] and promote inflammation [123]. Via regulation of macrophage functions, adipose-tissue-derived MPs facilitate foam cell formation, herein being central in the progression of atherosclerosis [124].

Depending on the donor cell and its state of activation, MPs express and transfer specific microRNAs (miRNA), which are thought to contribute to intercellular signalling [125,126,127,128,129]. miRNAs are single-stranded non-coding RNAs of up to 25 nucleotides, which bind to miRNA-response elements in untranslated regions of target genes, therefore regulating gene expression [130,131]. miRNA can also be found in plasma bound to proteins, such as argonaute 2 and high-density lipoprotein (HDL) [132,133].

Signalling via miRNA has been demonstrated to exert both pro- as well as anti-atherogenic effects on target cells and to regulate vascular inflammation, the formation of a neointima following stent implantation and endothelial regeneration [125,134,135]. In the context of cigarette smoking and PAD, the downregulation of miRNA-27b is independently associated with tobacco abuse and severity of PAD [128]. Following endovascular angioplasty and stent implantation for PAD, miRNA-195 has been found to predict adverse ischaemic events and the need for target vessel revascularisation [134].

In another study, miRNA-30c-5p was shown to inversely correlate with levels of LDL and plaque development, while miRNA-30c-5p expression in MPs was inhibited via the scavenger receptor CD36 by oxLDL and, in turn, modulated macrophage IL-1β release, caspase 3 and apoptosis [127]. Furthermore, miRNA-21 and miRNA-126 have also been independently associated with monocyte–platelet aggregate formation in acute coronary syndrome patients in vivo, as well as after TLR 1/2 activation [136]. In patients with CAD, MP miRNA enrichment and function was demonstrated to be impaired, which may contribute to disease progression [137]. Conversely, in an animal model of atherosclerosis, the incorporation of MPs of healthy controls resulted in improved EPC function due to miRNA transfer (miRNA-10a, miRNA-21, miRNA-126, miRNA-146a and miRNA-223) [138].

### 2.3. Neutrophil Extracellular Traps

Neutrophil extracellular traps (NETs)—web-like structures consisting of cell-free DNA—are extruded from neutrophils upon activation during inflammatory processes and consist of chromatin, histones and neutrophil granule proteins [139,140]. Previously, NETosis, which describes the process of neutrophils releasing NETs, was primarily regarded as a mechanism of the innate immune system to engulf and neutralise a wide range of extracellular pathogens including bacteria [139], viruses [141] and fungi [141]. However, NETosis is suggested to play a crucial role in inflammatory diseases including vasculitis [142], atherosclerosis and thrombosis [143].

There is increasing evidence that NETs contribute to endothelial dysfunction [144,145], glycocalyx degradation [9] and atherosclerosis [143] by generation of ROS and concomitant release of neutrophil granule proteins associated with atherogenesis, including neutrophil elastase and myeloperoxidase [146,147]. Vice versa, both enzymes also play a crucial role in the induction of NETosis [148,149]. Moreover, ROS stimulate the formation of pro-inflammatory MPs [150].

In atherosclerosis, oxLDL is also a potent stimulus for NET formation. Awasthi et al. have shown that incubation of neutrophils with oxLDL leads to NETosis in a time- and concentration-dependent manner [151]. OxLDL is likely to induce NETosis via TLR-2 and TLR-6, as their blockade resulted in significantly reduced NETosis [151]. Furthermore, the recognition of NETs promotes the production of an IL-1β precursor in macrophages and the subsequent release of mature IL-1β upon phagocytosis of oxLDL [152]. This, in turn, causes IL-17 production from T-cells [152]; IL-17 is a potent chemokine perpetuating the pro-atherogenic inflammatory environment [152]. In addition, oxidative stress induced by NET-associated enzymes, including myeloperoxidase and NO synthetase, is considered to promote oxidation of HDL, therefore rendering this inherently anti-atherosclerotic protein dysfunctional [153].

From a clinical perspective, NETs also offer relevant insight into the mechanisms of atherothrombosis [154,155]. Activated neutrophils and NETs were detected in about 90% of thrombi from patients with acute myocardial infarction and NET load correlated with infarct size and resolution of ST-segment elevation [155].

### 2.4. The Role of Inflammation in Aneurysm Formation

The most common location of aortic aneurysms is the infrarenal segment of the abdominal aorta [156]. While often asymptomatic, abdominal aortic aneurysms (AAA) are associated with significant mortality. In the UK, ruptured AAAs account for 7.5 and 3.7 deaths per 100.000 for men and women, respectively, while in the Mediterranean, these numbers are closer to 1.0–2.8 per 100.000 per year [157].

The presence of leukocytes [158,159], enzymes degrading ECM in the aortic wall [160,161] and excessive levels of inflammatory parameters [25] have been reported hallmarks of aneurysm formation. The risk factors associated with aneurysm formation are similar to those for atherosclerosis, namely, among others, male sex, dyslipidaemia and tobacco use [162,163].

While DM is a common risk factor for atherogenesis [22], it is associated with a reduction of morbidity due to AAA by almost a third [164]. DM enhances atherosclerosis progression and vascular calcification [165,166]. The latter accounts for a higher cardiovascular risk and higher mortality in diabetic patients and those with chronic kidney disease [167,168].

The observed survival benefit in diabetic patients with AAA is not yet fully elucidated and may be attributed towards DM itself or concomitant metformin therapy [169], as randomised placebo-controlled trials investigating metformin-repurposing for the prevention of AAA formation and enlargement are still ongoing [170,171,172]. Furthermore, increased vascular calcification is linked to aortic aneurysmal wall stabilization and slower AAA progression [173].

The estimated rate of comorbidity of atherosclerosis and aneurysm formation is about 27–53% [174,175]. Atherosclerosis and aneurysm formation are both increasingly regarded as inflammatory diseases, as leukocyte and platelet activation is a key factor for the pathogenesis of both disease entities [176,177,178]. AAA pathogenesis is characterised by infiltration of the aortic wall by neutrophils, macrophages and lymphocytes [179]. Subsequently, secreted enzymes, proteases and cytokines lead to ECM degradation, e.g., of collagen and elastin fibres, and an increased rate of apoptosis of smooth muscle cells promoting destruction and dilation of the vessel wall [180].

Macrophages are thought to play a decisive role in AAA formation [178]. Accumulation of macrophages during aneurysm formation can be observed in all three layers of the vessel wall but is particularly pronounced in the adventitia and the intraluminal thrombus (ILT) [181,182]. While the role of different subsets of macrophages in the stages of AAA development is not yet fully elucidated, it is hypothesised that bone-marrow-derived macrophages extravasate into the aortic wall and contribute to inflammatory processes and early stages of AAA formation [178].

The recruitment of monocytes into the aortic wall has been shown to be largely dependent on monocyte chemotactic protein 1 (MCP-1) and IL-6 produced by aortic adventitial fibroblasts [183]. Tieu et al. have shown that recruited monocytes locally mature into macrophages, which in turn stimulate the activation of adjacent fibroblasts and the release of further pro-inflammatory cytokines, forming a vicious circle of macrophage–fibroblast activation [183,184].

The pathways involved in AAA monocyte recruitment are also thought to play a decisive role in atherogenesis [185]. The infusion of angiotensin 2 in an apolipoprotein-E-deficient mouse model prone to atherosclerosis was not only shown to increase the severity of atherosclerotic lesions, but also promote AAA formation [186]. Upon stimulation by angiotensin 2, aortic adventitial fibroblasts release MCP-1 and IL-6, which cause monocyte recruitment and differentiation, and cytokine release [183,184].

The chemokine receptor 2 (CCR-2) signal, which is induced by MCP-1, plays a central role in various inflammatory diseases, including cancer and CVD [187]. Tieu et al. have demonstrated that the knock-out of CCR-2 resulted in significantly reduced adventitial fibroblast proliferation in a murine model of AAA formation [183]. Conversely, the transfer of CCR-2 positive monocytes resulted in restored proliferation and restored AAA formation [183]. The MCP-1/CCR-2 axis is thought to be crucial to the initiation of atherogenesis by promoting monocyte accumulation in atherosclerotic lesions [183,184]. In addition, levels of MCP-1/CCR-2 expression are associated with plaque vulnerability [188].

The activation of TLR-2 and TLR-4 and their downstream signalling pathways, including, among others, MyD88, NF-ĸB, and mitogen-activated protein kinase, is also considered a relevant driver of both aneurysm formation and atherosclerosis [34,189,190]. As a consequence, inhibition of the TLR-4/MyD88/NF-ĸB pathway by statins conveys anti-inflammatory and anti-atherosclerotic properties [21].

Neutrophils are considered to be both regulators and effector cells of inflammation [191]. In the context of AAA formation, activated neutrophils contribute to chronic inflammation, mainly by releasing ROS, NETs, histones and neutrophil granule proteins [192,193,194].

The formation of an ILT is frequently observed in progressive AAA and a risk factor for AAA rupture [195,196]. An ILT with concomitant platelet activation contributes to inflammation, vessel remodelling and ECM degradation [197]. Platelets activated in the context of ILT formation secrete pro-inflammatory cytokines and chemokines, which in turn stimulate leukocyte recruitment, activation and, ultimately, AAA progression [196,197,198,199].

Klopf et al. have reviewed various parameters including neutrophil-derived markers of inflammation, e.g., gelatinase-associated lipocalin [200,201], neutrophil elastase [202], myeloperoxidase [203,204], MMP [205] and NETs [206], as potential biomarkers for prognosis in AAA [25]. While the exact mechanisms which lead to aortic wall inflammation and leukocyte recruitment are not yet fully elucidated, these findings illustrate the involved processes and may help establish a better understanding of both factors determining prognosis and potential new therapeutic targets in AAA [25].

Importantly, inflammatory processes evoked by different infections, e.g., *Porphyromonas gingivalis*, *Epstein–Barr virus*, *cytomegalovirus* or *papillomavirus*, are also being discussed as potential promoters of local inflammation and risk factors for aneurysm formation [207,208]. In fact, the presence of periodontal disease, mainly with *Porphyromonas gingivalis* [209], and the occurrence of periodontal bacteria in the bloodstream or in the vascular lesion is associated with AAA formation [210,211,212]. In patients with AAA, *cytomegalovirus* was detected about five times as often as in healthy volunteers and was associated with increased levels of pro-inflammatory TNF-α and higher rates of arterial hypertension and CAD [208,213].

In addition to inflammatory conditions, aneurysms may also occur on the basis of pathogenic gene variants [214]. The variants best established generally concern structural proteins, e.g., procollagen type III α1, transforming growth factor β and fibrillin 1, as seen in Marfan syndrome [214].

### 2.5. Vasculitis

Vasculitides are a group of rare diseases characterised by auto-immune inflammation of blood vessels of various sizes [215]. The introduction of targeted immuno-modulatory agents has improved prognosis and reduced mortality due to exacerbated vasculitis or infection drastically [216]. In patients with vasculitis, CVD is now the most common cause of death [217,218]. In addition, a chronic inflammatory state is independently associated with long-term mortality in patients with Raynaud’s phenomenon [219].

An acceleration of atherogenesis in patients with AAV has been previously demonstrated [220], and surrogate markers of endothelial dysfunction, e.g., endothelium-dependent dilation of the brachial artery or pulse-wave velocity, are increased in the context of AAV [221]. One study evaluated atherosclerotic plaque burden by means of ultrasound and found that, compared to a healthy control cohort, AAV patients had a significantly higher plaque burden in the abdominal aorta and the carotid and the femoral arteries [220]. It may be hypothesised that a continuous sub-clinical inflammatory state contributes to the acceleration of atherogenesis in these patients [222]. The shedding of the endothelial glycocalyx, endothelial dysfunction [223] with enhanced expression of leukocyte adhesion factors, and leukocyte-diapedesis into the vessel wall promote a pro-inflammatory and pro-coagulatory state [224,225]. Furthermore, risk factors commonly associated with atherosclerosis are more prevalent in patients with AAV [223,226].

However, it must be noted that solid evidence of accelerated atherosclerosis in vasculitides has thus far only been established for Kawasaki’s disease, Takayasu’s arteritis and, most prominently, AAV [222].

Despite advances in immuno-modulatory therapy and the application of novel biologic disease-modifying drugs, glucocorticoids are still frequently used for induction therapy and are associated with significant toxicity. Traditional risk factors for atherosclerosis, i.e., hypertension, hyperglycaemia and dyslipidaemia, are exacerbated in patients with frequent glucocorticoid intake [227]. Risk factor management for the prevention of cardiovascular events in these high-risk patients has been shown to be insufficient in many patients [14,228]. Even with advanced biologics, e.g., Janus kinase inhibitors, undesired cardiovascular effects may occur [229,230]. Evidence is conflicting and therapeutic benefits may depend on the specific disease entity [231,232].

### 2.6. The Influence of Inflammation on Angiogenesis, Arteriogenesis, and Collateralisation

While inflammation is generally regarded as deleterious in PAD, specific inflammatory pathways involved in atherogenesis also participate in tissue regeneration, angio- and arteriogenesis [233,234]. Angiogenesis is the process of the formation of new capillaries for improved tissue perfusion, while arteriogenesis describes the transformation of arterio-arteriolar anastomoses to fully functional collateral arteries [235]. Therefore, while angiogenesis primarily involves endothelial cells, arteriogenesis necessitates the proliferation, migration and transformation of vascular smooth muscle cells [235], the latter being promoted by inflammatory conditions [233].

Angiogenesis is induced by various cytokines, e.g., vascular growth factor (VEGF), fibroblast growth factor (FGF) and angiopoietin, and is regulated via HIF-1 [87,236]. The molecular pathways which lead to arteriogenesis additionally include a response to increased shear stress and blood flow in arterio-arteriolar anastomoses and require the recruitment of macrophages [87].

The recruitment of macrophages is regulated via intercellular adhesion molecule 1 (ICAM-1) and CCR-2 signalling and promoted by granulocyte-colony stimulating factor (G-CSF) and granulocyte macrophage-colony stimulating factor (GM-CSF) [87,237,238]. While the M1 macrophage population is largely responsible for tissue damage associated with inflammation, alternatively activated M2 macrophages modulate cell proliferation and transition and are involved in tissue regeneration by secretion of growth factors (VEGF, FGF), MMPs and NO [87,239]. However, pro-inflammatory M1 macrophages are especially considered crucial sources of VEGF-A in arteriogenesis [234]. In this context, inflammatory M1 macrophages upregulate the transcription of the pro-angiogenic VEGF-A isoform via autocrine IL-1β-mediated activation of NF-ĸB and signal transducer and activator of transcription 3 (STAT3) [234]. Conversely, in an IL-1β knock-out mouse model, VEGF-transcription depending on HIF-1 alone was markedly decreased in comparison to a wild-type IL-1β cohort, where VEGF transcription is promoted by both HIF-1 and IL-1β-dependent pathways [234,240,241] (Table 1).

## 3. Current and Novel Therapeutic Targets and Strategies

Current guidelines in PAD emphasise metabolic risk management, including reduction of LDL levels, antiplatelet therapy, management of hypertension, glycaemic control, smoking cessation and physical activity [254,255,256,257,258]. While some of these interventions also exert a positive influence on systemic and local levels of inflammation [20,259,260], therapeutic strategies which directly intercept pro-inflammatory signalling pathways may be promising and are only beginning to be established [18]. In the following, we highlight some of the anti-inflammatory drugs and concepts. An overview of current and novel therapeutic targets and strategies is provided in Table 2.

### 3.1. Statins

The management of dyslipidaemia and, specifically, reduction of elevated levels of LDL has been a cornerstone of preventive cardiovascular medicine for many years. Statins, which inhibit hepatic 3-hydroxy-3-methylglutaryl-coenzyme-A reductase, and therefore impair cholesterol synthesis [261], are first line agents in treatment of CVD [18,254].

However, there is accumulating evidence that the positive effects of statins on atherosclerosis go beyond LDL reduction [262]. In fact, statin therapy has been demonstrated to result in increased endothelial biosynthesis of NO with a positive effect on vascular tone and platelet aggregation, and even plaque stabilisation or regression [262]. It has been suggested that statins also interfere with various endothelial adhesion molecules and therefore reduce leukocyte transmigration [263]. In patients with AAA, the anti-inflammatory properties of simvastatin treatment were shown by reduced TNF-α as well as cyclosporine A levels and a decreased amount of phosphorylated extracellular-signal regulated kinases (ERK) 1/2 [264,265]. Furthermore, a significant difference in the concentration of MMPs and their inhibitors was observed in aneurysmal wall tissue and ILT [266]. In addition, simvastatin reduced monocyte tissue factor expression in response to LPS treatment in healthy volunteers [267].

The anti-inflammatory effects of statins also include the reduction of c-reactive protein (CRP) concentration regardless of LDL levels [268]. Utilising fluorodeoxyglucose-positron emission tomography and computed tomography imaging, Tawakol et al. demonstrated a dose-dependent anti-inflammatory effect of atorvastatin in patients with suspected or proven atherosclerosis [269]. In a recent meta-analysis of three randomised controlled trials in patients receiving statins, inflammation, as assessed by CRP, was a stronger predictor than LDL for cardiovascular events and death [270].

Beyond CVD, the anti-inflammatory effects of statin therapy have also been demonstrated in other diseases. In chronic kidney disease, statins have resulted in lower levels of CRP [271], and in asthma, statins reduced both symptoms and biomarkers of inflammation [272].

### 3.2. Colchicine

Colchicine has been used for centuries for the treatment of inflammatory diseases, including gout and familial Mediterranean fever [17]. The pharmacodynamics of colchicine are complex, and they exert multiple effects on cellular signal transduction [17]. Colchicine has been demonstrated to reduce neutrophil chemotaxis by inhibition of the polymerisation of tubulin [273], reduce the expression of TNF-α [17,274], and attenuate the exocytosis of neutrophil granules [17,275]. Though less well elucidated, inhibitory effects of colchicine on the NLRP3 inflammasome have been observed and may inhibit the proliferation of smooth muscle cells as seen in the context of atherosclerosis [276].

The discovery of colchicine’s pleiotropic effects on inflammation and atherosclerosis [277,278] have led to the initiation of the phase III randomised placebo-controlled Colchicine Cardiovascular Outcomes Trial (COLCOT) [279]. Therein, 4745 patients who have suffered from myocardial infarction within the previous 30 months were randomised to receive either 0.5 mg of colchicine or placebo. The risk for the primary endpoints of cardiovascular death and serious cardiovascular events was reduced significantly in the colchicine group, with a hazard ratio (HR) of 0.77 [279].

Based on these findings, the 2021 guidelines on CVD prevention by the European Society of Cardiology have now included a class IIb, level A recommendation to consider low-dose colchicine in secondary prevention of CVD [18].

In the future, colchicine may also be applied in the context of acute myocardial infarction. A recent study by Wang et al. showed that an infusion of colchicine-loaded nanoparticles subsequent to myocardial infarction reduced inflammation and myocardial infarct size by 45% on average [280]. These findings highlight the potential of colchicine as a promising anti-inflammatory agent in CVD, both in the acute and chronic settings [281].

### 3.3. Eicosapentaenoic Acid Ethyl Ester

Eicosapentaenoic acid ethyl ester and its purified prescription form icosapent ethyl (IPE) is an omega-3 fatty acid and has demonstrated several anti-inflammatory and anti-atherosclerotic properties [282]. In a large, randomised placebo-controlled trial in patients with established CVD or several risk factors for the development of CVD and elevated triglyceride levels, the addition of IPE to standard statin treatment resulted in a highly significant risk reduction (HR 0.75) for ischaemic events and cardiovascular death when compared to placebo [283]. Furthermore, Budoff et al. documented a significant reduction in plaque size in patients with established CAD who received IPE when compared to a control group [284].

The exact mechanisms leading to these results remain to be established, especially as allocation to the IPE cohort in the Reduction of Cardiovascular Events with EPA—Intervention Trial (REDUCE-IT) did not result in a relevant reduction of inflammatory parameters [283]. In other trials, however, a high-sensitivity CRP and lipoprotein-associated phospholipase A2 lowering effect has been documented [282].

It is hypothesised that protective effects with regard to CVD may be attributed to the production of the bioactive IPE metabolites thromboxane A3 and prostacyclin, which exert antithrombotic influence on platelets and promote endothelial vasodilation [285]. In addition, IPE integration in cellular membranes also seems to have a biophysical anti-atherogenic effect [285].

The reduction in TLP, which is observed under large doses of IPE, is also considered to have protective effects in CVD [286]. As these predominantly transport saturated fatty acids, which are thought to promote activation of the NLRP3 inflammasome, the reduction of TLP levels may also attenuate atherogenesis [285,287].

### 3.4. Canakinumab and Anakinra

The NLRP3 inflammasome is a pro-inflammatory signalling complex with pleotropic effects on cytokine release and cleavage of pro-interleukins [61]. Its activation is mediated by pathogen- and damage-associated molecular patterns including, among others, TLR-2 and TLR-4, and results in activation of the IL-1 pathway [61]. In the context of atherogenesis, the NLRP3 inflammasome is activated by the recognition of oxLDL and cholesterol crystals via various receptors in macrophages. The subsequent release and formation of, among others, IL-1β results in activation of endothelial cells, promotes the expression of adhesion molecules and the proliferation of smooth muscle cells and increases the production of MCP-1 [61].

The monoclonal antibody canakinumab, which targets IL-1β, and anakinra, an IL-1β receptor antagonist, are applied in various immune-mediated disorders [288] and were also considered potential therapeutics in atherosclerosis [289,290,291,292]. The pivotal Antiinflammatory Therapy with Canakinumab for Atherosclerotic Disease (CANTOS) trial has demonstrated a dose-dependent effect of canakinumab on systemic levels of inflammation in patients with previous myocardial infarction [15]. In this trial, 150 mg of canakinumab every three months reduced the risk of adverse cardiovascular events (HR: 0.85) compared to placebo, independently of lipid level lowering [15].

### 3.5. Glucocorticoids

Glucocorticoids are analogues of endogenous cortisone and constitute a cornerstone of the treatment of various chronic inflammatory conditions [293]. Glucocorticoids exert their pleiotropic effects by binding to intracellular steroid-receptor proteins and regulate gene expression and cellular signalling [294].

However, long-term glucocorticoid excess is also associated with significant undesirable effects including hyperglycaemia [295], arterial hypertension [296], obesity [297], dyslipidaemia [298] and dysregulation of the coagulation cascade [299], all of which are considered well-established risk factors for atherogenesis and adverse cardiovascular events [294]. In patients diagnosed with Cushing’s disease, which is characterised by endogenous overproduction of cortisone, a thickened intimal-medial layer and a lower systolic carotid artery lumen diameter [300] have been observed [301]. Furthermore, the ankle-brachial pressure index is elevated in Cushing’s disease [302]. In large register studies, glucocorticoid prescription has been associated with significant and dose-dependent increases in the risk for CVD [301,303,304,305].

### 3.6. Antidiabetic Drugs

Several antidiabetic drugs have been proposed to promote anti-inflammatory pathways or inhibit pathways associated with inflammation [306].

Sodium-glucose cotransporter 2 (SGLT-2) inhibitors and glucagon-like peptide 1 (GLP-1) receptor agonists were originally developed for the control of hyperglycaemia in patients with DM type 2. SGLT-2 inhibitors prevent glucose reabsorption in the proximal tubule and cause glucosuria, therefore lowering glucose levels in serum [307].

Large-scale clinical trials have demonstrated that SGLT-2 inhibitors significantly reduce the risk for hospitalisation and cardiovascular death in patients with heart failure [308,309,310,311]. Today, SGLT-2 inhibitors constitute an integral component of heart failure therapy and are recommended in the most recent heart failure guidelines [312]. While the detailed molecular mechanisms of the observed clinical benefits are largely unknown, recent studies have proposed potential anti-inflammatory properties of these substances in addition to pleiotropic, metabolic and cardiovascular effects of SGLT-2 inhibitors, which have been described in detail by Hou et al. [313,314].

In murine models, SGLT-2 inhibitors were demonstrated to reduce the expression of MCP-1 and IL-1β [315,316,317]. A reduction of inflammasome activation and the subsequent release of IL-1β was also found in patients with DM [318]. Furthermore, macrophage behaviour seems to be influenced by SGLT-2 inhibitors as increased autophagy and cholesterol efflux were observed in a murine model. Therein, the modulation of an AMPK-dependent pathway resulted in attenuated atherosclerosis [319]. Among a plethora of metabolic effects, GLP-1 receptor agonists were also found to exert a robust anti-inflammatory effect by lowering the levels of ROS generation and reducing NF-ĸB activation, as well as expression of mRNA coding for, among others, TNF-α, IL-1β, TLR-2 and TLR-4 [320,321]. In apolipoprotein-E- and LDL-receptor-deficient mice, the application of liraglutide or semaglutide resulted in decreased aortic intima thickening and inhibited plaque progression compared to a control group [322]. Semaglutide was also demonstrated to alter the expression of genes associated with inflammation and atherogenesis including IL-6, chemokine ligand 2, MMPs and proteins relevant for cholesterol metabolism [322]. In vitro, GLP-1 receptor agonists have been shown to modulate macrophage behaviour and reduce the secretion of pro-inflammatory cytokines (e.g., interferon γ, TNF-β, IL-1β, IL-2 and IL-6), and promote the release of anti-inflammatory IL-10 [323].

Clinical trials in patients with DM type 2 showed a consistent reduction in CRP, TNF-α and malondialdehyde [324]. Cardiovascular outcomes were also improved in patients with DM type 2 receiving GLP-1 receptor agonist treatment in some, but not all, clinical trials [325]. In a retrospective cohort study of patients with PAD and DM under either SGLT-2 inhibitors, GLP-1 receptor agonists or sulfonylureas, GLP-1 receptor agonist prescription was associated with a significantly lower rate of lower limb amputation [326]. Metformin used to be considered the established first-line therapy for patients with DM type 2 for decades [327]. In recent years, the attention has been increasingly focused on the effects of metformin beyond control of hyperglycaemia [328]. Though the exact pharmacodynamic properties of metformin have yet to be fully elucidated, there is some mechanistic evidence that metformin may attenuate atherogenesis [329,330]. In vitro studies have demonstrated that metformin attenuates foam cell formation and phagocytosis of oxLDL [331]. On a molecular level, metformin administration resulted in reduced expression of the macrophage scavenger receptor A and CD36, both of which are involved in oxLDL uptake [242,243,331]. The expression of inflammatory markers, including IL-1β, IL-18, cysteinyl aspartate specific proteinase-1, NLRP3 and ROS, was reduced in macrophages treated with metformin [331]. In a rabbit model of atherosclerosis, treatment with metformin resulted in significantly decreased burden of atherosclerotic lesions with lower macrophage content [332]. In addition to reducing plasma levels of MCP-1, CRP and TNF-α, metformin also reduced the expression of mRNA coding for vascular adhesion molecule 1 and intercellular adhesion molecule 1, therefore ameliorating adhesion of monocytes to endothelial cells [332]. Furthermore, the activation of AMPK, the inhibition of NF-ĸB expression and NET formation are also considered potentially anti-atherogenic properties of metformin [329,333,334].

Though there is some evidence that metformin therapy has a positive effect on cardiovascular outcomes in patients with DM type 2 [329,330,335], the data are hitherto contradictory for non-diabetic patients [336,337,338,339]. The ongoing Glucose Lowering in Non-diabetic hyperglycaemia trial (GLINT) [340,341] may help to determine the role of metformin in prevention of CVD in these patients [339].

Dipeptidyl peptidase 4 (DPP4) inhibitors or gliptins are established second-line anti-diabetic agents which exert their effect by inhibition of proteolysis of endogenous GLP-1 and glucose-dependent insulinotropic polypeptide [342,343].

DPP4 is involved in the cleavage of chemokines and cytokines, therefore potentially exhibiting a role in cell–cell communication [344]. Furthermore, it is suggested that DPP4 induces endothelial dysfunction and promotes the expression of TLR-2 and TLR-4 and subsequent activation of inflammatory pathways [344,345,346]. The inhibition of DPP4 is therefore considered to attenuate inflammation and improve endothelial function, potentially by stimulation of NO synthesis and reduction of endothelin 1 expression [344,347,348,349]. Gliptins have also been demonstrated to reduce the expression of vascular adhesion molecules and MCP-1, TNF-α, IL-1β and IL-6, as well as LDL- or lipopolysaccharide-induced foam cell formation, most likely due to attenuation of NF-ĸB and c-Jun N-terminal kinase signalling and AMPK phosphorylation [344,347]. There is evidence that DPP4 inhibitors can increase the number of circulating EPCs [349,350,351] and repress the activation of the NLRP3 inflammasome [349,352]. On a systemic level, reduced hepatic production of TLP [353] and an accelerated postprandial lipid metabolism [354] have been observed under DPP4 therapy [344].

While pre-clinical data may look promising and gliptins have been demonstrated to reduce established risk factors for CVD in patients with DM type 2, including dyslipidaemia and hypertension [344], randomised controlled trials have thus far failed to demonstrate a beneficial effect beyond glycaemic control with regard to cardiovascular outcomes [343,355,356,357,358].

**Table 2:** Potential novel applications of established therapeutic agents.

	Standard Application	Proposed Mechanism	Clinical Effect	Selected Evidence
Statins	LDL reduction secondary prevention of CVD	NO synthesis ↑ leukocyte adhesion ↓	cardiovascular events and death ↓	Tawakol et al. [269] Ridker et al. [270]
Colchicine	gout familial Mediterranean fever	leukocyte chemotaxis ↓ TNF-α ↓ exocytosis of neutrophil granules ↓ NLRP3 activation ↓	cardiovascular events and death following MI ↓	Tardif et al. [279] Chen et al. [281]
Icosapent ethyl	no previous application	active metabolites (thromboxane A3, prostacyclin) ↑ biophysical effect on cell membranes TLP ↓	cardiovascular events and death in established CVD or risk for CVD and hypertriglyceridemia plaque progression ↓	Bhatt et al. [283] Budoff et al. [284]
Glucocorticoids	various inflammatory conditions	modulation of gene transcription	risk of CVD including CAD, PAD ↑	Pujades-Rodriguez et al. [305] Macleod et al. [301]
IL-1β antagonists	cryopyrin-associated periodic syndromes gout familial Mediterranean fever macrophage activation syndrome recurrent pericarditis rheumatoid arthritis systemic juvenile idiopathic arthritis	endothelial activation ↓ adhesion molecule expression ↓ smooth muscle cell proliferation ↓ MCP-1 ↓	cardiovascular events and death in patients with elevated CRP and MI	Ridker et al. [15]
SGLT-2 inhibitors	DM type 2	NLRP3/IL-1β/MCP-1 pathway ↓ AMPK pathway ↑ cholesterol efflux and autophagy in macrophages ↑	hospitalization and cardiovascular death in heart failure	McMurray et al. [308] Solomon et al. [359] Packer et al. [360] Anker et al. [311]
GLP-1 receptor agonists	DM type 2	ROS generation ↓ NF-ĸB activation ↓ INF-γ, MMP, TNF-β, IL-1β, IL-2, IL-6 from macrophages ↓ IL-10 ↑	CRP, TNF-α ↓ Trials inconclusive	Bethel et al. [325]
Metformin	DM type 2	oxLDL phagocytosis ↓ scavenger receptor A, CD36 ↓ NLRP3, ROS, MCP-1, CRP, TNF-α NET formation ↓ NF-ĸB activation ↓ AMPK pathway ↑	all-cause death in DM type 2 and atherothrombosis↓	Roussel et al. [335] GLINT (ongoing) [340,341]
DDP4 inhibitors	DM type 2	NO synthesis ↑ endothelin 1 ↓ MCP-1, TNF-α, IL-1β, IL-6 ↓ NF-ĸB activation ↓ AMPK and c-Jun N-terminal kinase pathway ↑ NLRP3 activation ↓ TLP ↓	dyslipidaemia and hypertension in patients with DM type 2 ↓ cardiovascular death, MI, stroke in patients with DM type 2 ~	Rosenstock et al. [355] Green et al. [356] Scirica et al. [357] White et al. [358]

Abbreviations: AMPK, adenosine-monophosphate-kinase; CAD, coronary artery disease; CD, cluster of differentiation; CRP, c-reactive protein; CVD, cardiovascular disease; IL, interleukin; INF, interferon; LDL, low-density lipoprotein; MCP-1, monocyte chemotactic protein 1; MI, myocardial infarction; NF-ĸB, nuclear factor kappa B; NLRP3, nucleotide oligomerization domain, leucine-rich repeat, and pyrin domain-containing protein 3; NO, nitric oxide; oxLDL, oxidised low-density lipoprotein; PAD, peripheral artery disease; ROS, reactive oxygen species; TLP, triglyceride-rich lipoprotein; TNF, tumour necrosis factor; ↑, increase in expression or function; ↓ decrease in expression or function; ~, no or inconclusive effect.

### 3.7. Antiplatelet Therapy

Current antiplatelet regimes interfere with thromboinflammatory pathways; however, platelet reactivity is, to a wide extent, also determined by alternative platelet activation pathways despite adequate guideline-driven platelet inhibition [255,361,362,363,364]. Platelet activation and the formation of platelet–leukocyte aggregates is a hallmark of inflammatory atherosclerotic processes [198].

Recently, there is increasing evidence that platelet-to-lymphocyte ratio (PLR)—a simple marker calculated from the blood count—is related to platelet activation and ischemic events in CVD [198,365,366,367]. Moreover, a high PLR is also related to target vessel restenosis after revascularization in PAD [368].

Platelet reactivity can be modulated by various conditions such as age [369], sex [370], HDL levels [371,372] and cytochrome P450 2C9/2C19 polymorphism [373,374], but also anaemia [375,376]. The latter is often associated with chronic inflammation and implicated in both thrombotic and bleeding events [375,377]. Moreover, iron deficiency is associated with major adverse cardiovascular and leg events in PAD, suggesting anaemia as a possible therapeutic target [378].

Another aspect relevant for pain management of PAD patients with critical ischaemia is the drug interaction of morphine or fentanyl, as a decrease in plasma levels and/or antiplatelet effects of P2Y12 inhibitors can occur [379,380,381,382]. In contrast, morphine did not exert a significant effect on aspirin-mediated platelet inhibition [383].

Platelet activation, furthermore, has a high impact on platelet metabolism and redox balance [384]; hence, attenuation of platelet reactivity may have beneficial effects on redox processes.

### 3.8. Attenuation of Ischaemia-Reperfusion Injury

The counter regulation of the decrease in NO bioavailability due to I/R injury is one possible therapeutic approach to minimize endothelial dysfunction. Herein, dietary supplementation of NO donors, enhancers of NO availability, NO synthase inducers and antioxidants have been studied [385,386].

Interestingly, pleiotropic effects of statins include the increase in endothelial NO synthase expression and function [387]. In patients with AAAs, simvastatin reduced lipid peroxidation level as demonstrated by lower 4-hydroxy-trans-2-nonenal concentration [265]. Moreover, simvastatin has been shown to induce heme oxygenase 1 (HO-1), an enzyme with anti-inflammatory, antioxidant, antithrombotic, pro-angiogenetic and antiapoptotic properties [388,389,390,391]. Induction of HO-1 can also be achieved by heme arginate infusion, which improves reperfusion patterns during I/R injury [392,393,394].

Further concepts to ameliorate I/R injury include a plethora of therapies, such as blocking of intercellular adhesion molecule 1, administration of polymerised albumin, colchicine, tocilizumab, anakinra and revacept and pre-, per- and postconditioning [395,396,397,398,399,400]. Exercise-induced I/R injury in PAD can also be attenuated using cilostazol, which results in a reduced expression of P-selectin, intercellular adhesion molecule 1 (ICAM-1) and vascular cell adhesion molecule 1 (VCAM-1) [401]. P-selectin and ICAM-1, as well as VCAM-1, are known to promote leukocyte recruitment to sites of inflammation and are regulated via NF-ĸB [402,403,404]. In addition, a double-blind randomised controlled trial of cilostazol in patients with PAD has demonstrated marked effects on EPC function, which may improve collateral vessel formation [405]. The homing of EPCs can be enhanced by SDF-1, which binds to CXCR-4 and has shown to improve ischemic tissue perfusion and increase capillary density in mice [88]. However, in the STOP-PAD trial (SDF-1 plasmid Treatment for Patients with Peripheral Artery Disease; a randomized, double-blind, placebo-controlled clinical trial), the injection of JVS-100, a non-viral DNA plasmid-based therapy encoding SDF-1, did not improve hemodynamic measures or wound healing at 3 months [406]. Another important cornerstone in the therapy of risk factors in PAD patients are angiotensin-converting enzyme inhibitors, which ameliorate (micro-)vessel perfusion by increasing nitric oxide production [407]. In addition, ROS formation is, amongst others, reduced by SGLT-2 inhibitors and GLP-1 receptor agonists [320,408,409]. One of the underlying mechanisms might be the reduction of succinate levels by SGLT-2 inhibition; however, on the contrary, the GLP-1 receptor agonist liraglutide elevates succinate levels despite ameliorating mitochondrial function [410,411].

### 3.9. Vascular Regeneration

The interactions between molecular signalling pathways involved in both inflammation and vascular regeneration imply a potential for therapeutic modulation. Different approaches tested so far include the application of GM-CSF [412], basic FGF [413] and plasmid-based SDF-1 gene therapy [406]; however, clinicals trials were either negative or terminated prematurely due to adverse events largely attributed to inflammatory reactions. Novel application systems, including peptide-loaded microgels and microspheres, are hypothesised to help overcome inflammation-associated effects [414,415]. However, so far, these have been explored in murine models only [414,415].

The homing and angiogenic function of EPCs has been demonstrated to be modulated by inflammatory processes and can be improved by the inhibition of macrophage inflammatory protein-1β (MIP-1ß) [416]. This is especially relevant in the context of DM, where mononuclear cells and EPCs exhibit increased MIP-1β secretion and consecutively impaired expression of VEGF, SDF-1α and other pro-angiogenetic cytokines [416].

Moreover, the abundance of cytokines during ischaemia is suggested to create an environmental condition facilitating transdifferentiation of fibroblasts into endothelial cells [87]. This is also promoted by an increased accessibility of DNA in the context of injury and ischaemia mediated by NF-ĸB activation, a process referred to as transflammation [417]. Herein, therapeutic interaction could be conferred by modification of TLR signalling pathways, as shown for TLR-3 agonism in combination with EC growth factors, that result in fibroblast transdifferentiation into endothelial cells [418].

The modulation of macrophage polarisation and behaviour is also hypothesised to be a critical determinant and potential target following endovascular procedures for PAD [419]. An intervention to boost macrophage M2 polarisation may help to support endothelial repair by the release of proangiogenic signalling molecules, i.e., basic FGF-2, VEGF-A and transforming growth factor (TGF-)β [419]. This could ultimately improve outcomes following endovascular interventions in PAD [419].

Further studies on possible therapeutic concepts for the modulation of transdifferentiation, transflammation and vascular regeneration are warranted [87].

### 3.10. Physical Exercise

Regular physical exercise is a cornerstone in the prevention of CVD [18]. Besides improving endothelial function by increasing circulating EPC numbers [420], low intensity aerobic training also increases capillary density in skeletal muscle [421]. Furthermore, significant positive effects on established cardiovascular risk factors, e.g., hyperglycaemia [422], hypertension [423] and dyslipidaemia [421], have been demonstrated and a reduction in systemic markers of inflammation can be observed with physical exercise [424]. These include, among others, TNF-α and CRP, as well as the expression of vascular adhesion molecules, all of which are considered to be of crucial importance in the pathogenesis of atherosclerosis [424]. In addition, protective effects of previous physical activity may also improve outcomes following cardiovascular events [425,426].

## 4. Discussion

PAD is increasingly regarded as an inflammatory process affecting not only the macro- but also the microvasculature [10,255,258]. Herein, modification of glycocalyx conformation, charges and density leads to endothelial dysfunction [44]. Thromboinflammatory processes involving leukocyte and platelet activation, as well as ETosis, are central pathomechanisms in plaque formation [43,44].

Altered flow conditions due to plaque formation promote further disease progression by modulation of endothelial cell metabolism [427]. Upregulated 6-phosphofructokinase/2,6-bisphosphatase 3 (PFKFB3), which is a key enzyme in endothelial glycolysis, gives an impulse for angiogenesis with immature vessel formation, thus enhancing plaque vulnerability [427,428,429]. Moreover, rupture of the atheroma followed by atherothrombosis may also be triggered by (N)ETosis, as neutrophil, macrophage and mast cell activation play a critical role in atherosclerotic lesions [43,430,431].

In addition, NETs were shown to contribute to fibrous vascular occlusion [154]. This may also contribute to systemic microvessel rarefication, which was observed in PAD and other CVDs [432,433,434,435].

**Figure 1:**
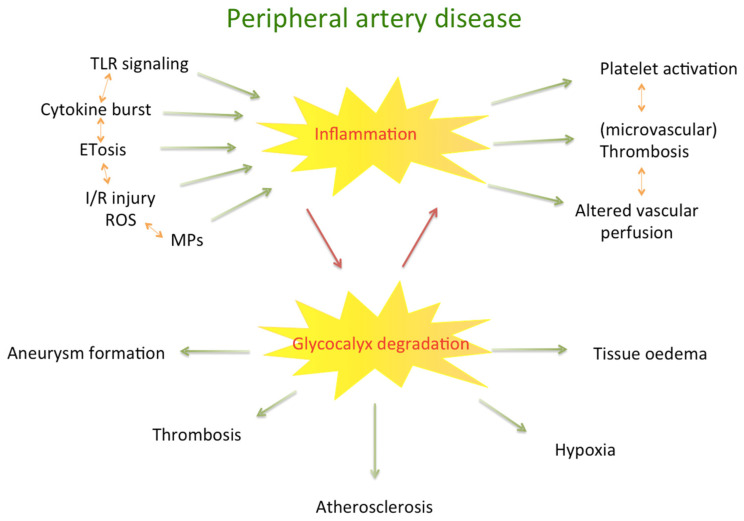
Pathophysiologic consequences of inflammation on the vasculature and adjacent tissue: Inflammatory processes promotes endothelial dysfunction by glycocalyx degradation, leading to altered vascular homeostasis [9]. The pathological processes influence each other, perpetuating disease progression. ETosis, extracellular trap formation; I/R, ischaemia-reperfusion injury; MPs, microparticles; ROS, reactive oxygen species; TLR, toll-like receptor.

NET release promoting subsequent microvascular thrombosis is regarded as a hallmark of atherosclerotic processes. The interplay of platelet activation, platelet–leukocyte aggregate formation, ETosis and ROS formation perpetuates thromboinflammation, resulting in altered microvascular fluid filtration, microthrombosis and finally, tissue necrosis (compare Figure 1) [9,11].

Capillary perfusion is also impaired by ROS formation during I/R injury, as it occurs during ischemic vascular diseases [11]. I/R injury also contributes to postischemic capillary no-reflow after successful arterial recanalization [11]. Attenuation of I/R injury to preserve microvascular haemodynamics [399] will be of importance for refinement of interventional PAD treatment.

Acute critical ischemia of the lower extremity often occurs in the setting of a total vessel occlusion, yet PAD patients encompass a wide spectrum of disease, including chronic (and often asymptomatic) disease courses, with progressive atherosclerosis, and development of collateral circuits [436]. In addition, it is also known that patients with lower extremity PAD are at higher risk of ischaemic events than those patients with isolated coronary artery disease [437]. Moreover, chronic inflammation, together with pro-thrombotic stimuli, re-endothelialisation, vascular smooth muscle cell migration and proliferation, as well as matrix remodelling, account for the limited patency of vascular stents and bypass grafts, herein presenting as a wider disease pathomechanism than the initial atherosclerotic lesion [438].

Inflammatory pathomechanisms might also differ during acute and chronic PAD processes. Herein, it should be emphasized that even different symptomatic PAD subpopulations, with regard to PAD severity and comorbidities, can experience a difference in therapeutic benefits [439]. The latter has been shown in the subanalysis of the COMPASS trial, where the combination therapy of aspirin and low-dose rivaroxaban conferred the highest estimated absolute risk reduction at 30 months in those patients with high-risk limb presentation or high-risk comorbidity at baseline [439]. Moreover, the results of the COMPASS trial also showed a greater reduction in major adverse limb events (MALE) in PAD patients with a high-risk limb presentation and a greater reduction in major adverse cardiac events (MACE) in PAD patients with high-risk comorbidity [439].

In the context of the SARS-CoV-2 pandemic and its long-term consequences, it should be noted that viral persistence promoting (subclinical) inflammation will have an impact on the vasculature and atherosclerosis [9,440,441,442]. The detection of (subclinical) inflammation has recently been also proposed through measurements of cholinesterase levels, which are declined during inflammation and also linked to patient mortality [443,444]. In PAD patients, low levels of serum cholinesterase were associated with long-term adverse ischemic events after angioplasty and stenting of the superficial femoral artery [445]. However, more studies, in particular in comparison to established inflammatory markers, are needed.

In addition, it should be noted that inflammation does not only confer a physical limitation through atherosclerotic processes, but also impacts the patient’s mood and personality functioning and can result in the development of vascular depression in patients with cardiovascular diseases [446].

Despite all pharmacotherapeutic progress, modulation of the fragile glycocalyx and, in consequence, preservation of endothelial cell function is demanding. Concepts to reduce endothelial dysfunction by interference with redox processes have hitherto only marginally been integrated into clinical practice [447]. However, different pharmacotherapies, such as statins, angiotensin converting enzyme inhibitors, GLP-1 receptor agonists or SGLT-2 inhibitors, may ameliorate inflammatory pathways [261,314,321,448]. The reduction of fibrosis and arterial stiffness may directly influence long-term pathogenesis [449].

While the therapeutic promotion of arteriogenesis and angiogenesis may be a promising concept to alleviate signs and symptoms of PAD, the complex interactions with inflammation complicate effective therapeutic applications so far. As it seems, some level of inflammation is required for effective neovascularisation; this is also observed in atherosclerotic plaques, where neovascularisation constitutes a hallmark of progression and plaque vulnerability [450].

In addition to pharmacotherapy, exercise training should remain a cornerstone in patients with stable PAD [18]. In particular, aerobic exercise training has been shown to upregulate microvessel perfusion [451]. Moreover, the number of circulating EPCs increases in patients with regular endurance training and is associated with improved endothelial function [420,452]. Therefore, future concepts should emphasize preventive strategies [18] including governmental-promoted exercise training and programs to raise awareness for cardiovascular risk factors.

One limitation of our review is the scarce evidence from randomised controlled clinical trials in patients with PAD; therefore, additional knowledge on inflammatory concepts is derived from animal studies and trials in patients with other vascular disease entities, where pathomechanisms might differ. However, where available, the literature regarding PAD is presented primarily. Moreover, in this review, we also wanted to highlight the common share of vascular diseases, including aneurysms, namely inflammatory processes leading to endothelial dysfunction. Further clinical trials are needed to give more insights into potentially unique local inflammatory pathomechanisms, which may be different throughout PAD stages and during acute and chronic ischaemic processes.

## 5. Conclusions

Inflammatory pathways have a critical role in the development, disease perpetuation and complications of atherosclerosis. Novel research results regarding disease pathophysiology imply the need for a paradigm shift in the therapeutic approach to atherosclerotic diseases. In the future, the attenuation of (subclinical) inflammatory processes will become equally important to other risk-factor management in the therapy of PAD. However, further studies regarding the long-lasting outcomes of PAD patients are warranted.

## Figures and Tables

**Table 1:** Triggers of inflammation in peripheral artery disease.

	Triggers	Involved Pathways	Resultant Effects
Endothelial dysfunction	glycocalyx degradation [26] ROS [11] ETs [144,145]	eNOS ↓ [105] TLR/MyD88/MAPK/NF-ĸB ↑ [34] thrombin/PAR-1 and 4 ↑ [46]	endothelial permeability ↑ [30] leukocyte rolling and diapedesis ↑ [28,107] platelet adhesion and activation ↑ [29] binding of anticoagulant mediators ↓ [27] endothelium-induced vasodilation ↓ [120,121] macrophage M1 polarisation ↑ [83]
(Oxidised) LDL accumulation	endothelial dysfunction and glycocalyx degradation [32] hyperlipidaemia [3]	scavenger receptor A [242,243] TLR-2 and -6/MyD88/MAPK/NF-ĸB ↑ [151] NLRP3/IL-1β ↑ [61,152] PKC/IRAK/MAPK ↑ [151]	NET formation ↑ [151] ROS ↑ [151] endothelial dysfunction ↑ [61] SMC proliferation ↑ [61] leukocyte recruitment ↑ [61] MCP-1 ↑ [61]
Oxidative stress and ROS	I/R injury [10] microparticles [120,121] NETs [146,147] NF-ĸB [244] AGE [10]	NF-ĸB ↑ [244] PPARγ/AMPK/eNOS ↓ [245,246,247] NLRP3/IL-1β ↑ [11] adiponectin ↓ [247,248,249,250]	endothelial dysfunction ↑ [11,110] ET and MP formation ↑ [91,150] SMC proliferation ↑ [251] ICAM-1, VCAM-1 ↑ [252]
Ischaemia and reperfusion	Impaired perfusion and resolution	TCA dysfunction with succinate accumulation [79] HIF-1α/IL-1β ↑ [80] NO bioavailability ↓ [78] ROS ↑ [82] EPC function ↓ [85]	ROS ↑ [11] macrophage M1 polarisation ↑ [83] microvascular perfusion ↓ [11] regenerative potential ↓ [85]
TLR activation	oxLDL [151] DAMPs (oxLDL, HSP, fibronectin, fibrinogen, hyaluronic acid derivates) [34,189] PAMPs (bacterial, viral and fungal) [34,189]	caspase 3 ↑ [85,90] TLR/MyD88/MAPK/NF-ĸB ↑ [34] NLRP3/IL-1β ↑ [34]	EPC apoptosis ↑ [85,90] endothelial dysfunction ↑ [61] SMC proliferation ↑ [61] leukocyte recruitment ↑ [61] MCP-1 ↑ [61] aneurysm formation ↑ [253]

Abbreviations: AGE, advanced glycation end-products; AMPK, adenosine-monophosphate-kinase; DAMPs, danger-associated molecular patterns; eNOS, endothelial nitric oxide synthetase; EPC, endothelial progenitor cell; ET, extracellular trap; HIF-1α, hypoxia-inducible factor 1α; HSP, heat-shock protein; I/R, ischaemia-reperfusion injury; ICAM-1, intercellular adhesion molecule 1; IL-1β, interleukin 1β; IRAK, interleukin 1 receptor-associated kinase; MAPK, mitogen-activated protein kinase; MCP-1, monocyte chemotactic protein 1; MP, microparticle; MyD88, myeloid differentiation factor 88; NETs, neutrophil extracellular traps; NF-ĸB, nuclear factor kappa B; NLRP3, nucleotide oligomerization domain, leucine-rich repeat, and pyrin domain-containing protein 3; oxLDL, oxidised low-density lipoprotein; PAMPs, pathogen-associated molecular patterns; PAR, protease-activated receptor; PKC, protein kinase C; PPARγ, peroxisome proliferator-activated receptor γ; ROS, reactive-oxygen species; SMC, smooth muscle cell; TCA, citric acid cycle; TLR, toll-like receptor; VCAM-1, vascular adhesion molecule 1; ↑, increase in expression or function; ↓ decrease in expression or function. Please note that TLR-signalling involved in immunothrombosis is complex and has many branching paths; therefore, we only depicted the predominantly used pathway [34].
